# Etoposide treatment in secondary hemophagocytic syndrome: impact on healthcare-associated infections and survival

**DOI:** 10.1186/s13613-022-01075-9

**Published:** 2022-10-28

**Authors:** Thibault Dupont, Michael Darmon, Eric Mariotte, Virginie Lemiale, Jehane Fadlallah, Adrien Mirouse, Lara Zafrani, Elie Azoulay, Sandrine Valade

**Affiliations:** 1 Assistance Publique-Hôpitaux de Paris (APHP), Medical Intensive Care Unit, Saint-Louis University Hospital, 1 Avenue Claude Vellefaux, 75010 Paris, France; 2 Université de Paris, Paris, France; 3 UMR 1153, Center of Epidemiology and Biostatistics, ECSTRA Team and Clinical Epidemiology, Sorbonne Paris Cité, CRESS, INSERM, Paris, France; 4 Assistance Publique-Hôpitaux de Paris (APHP), Immunology Department, Saint-Louis University Hospital, Paris, France

**Keywords:** Hemophagocytic syndrome, Etoposide, Hospital acquired infections

## Abstract

**Background:**

Etoposide remains the cornerstone of symptomatic management of critically ill patients with secondary hemophagocytic syndrome (sHS). Risk of healthcare-associated infections (HAIs) in this setting with etoposide has never been assessed. We sought to evaluate the association between etoposide administration, HAIs occurrence and survival in critically ill adult patients with sHS. In this retrospective single-center study conducted in a university hospital ICU between January 2007 and March 2020, all consecutive patients with sHS were included. HAIs were defined as any microbiologically documented infection throughout ICU stay. Competing risk survival analysis was performed to determine factors associated with HAIs. Propensity score-based overlap weighting was performed to adjust for factors associated with etoposide use.

**Results:**

168 patients with a median age of 49 [38, 59] were included. Forty-three (25.6%) patients presented with at least 1 microbiologically documented HAI throughout ICU stay. After adjustment, cumulative incidence of HAI was higher in patients receiving etoposide (*p* = 0.007), while survival was unaffected by etoposide status (*p* = 0.824). By multivariable analysis, etoposide treatment was associated with a higher incidence of HAIs (sHR 3.75 [1.05, 6.67]), whereas no association with survival (sHR 0.53 [0.20, 1.98]) was found. Other factors associated with increased mortality after adjustment included age, immunodepression, male sex, SOFA score > 13, and occurrence of HAI.

**Conclusions:**

In patients with sHS, etoposide treatment is independently associated with increased occurrence of HAIs, whereas no association with survival was found. Intensivists should be aware of increased infectious risk, to promptly detect and treat infections in this specific setting. Studies to assess benefits from prophylactic anti-infectious agents in this setting are warranted and the lack of benefit of etoposide on survival needs to be interpreted cautiously.

**Supplementary Information:**

The online version contains supplementary material available at 10.1186/s13613-022-01075-9.

## Background

Hemophagocytic syndrome (HS), or hemophagocytic lymphohistiocytosis (HLH) is a rare but life-threatening condition, with high overall mortality rates in the ICU [1]. The pathophysiology relies on defective cytotoxicity in T-CD8 lymphocytes and NK-cells [2], resulting in uncontrolled macrophage activation and cytokine release after antigen stimulation, leading to multiple organ dysfunction [3–7]. HS is traditionally characterized as being either familial (fHS) or secondary (sHS), the latter being most prevalent in the setting of an adult ICU population, resulting from infectious (viral, bacterial and parasitic) and non-infectious triggers (malignancy, autoimmune disease), with underlying immune suppression [1]. Diagnosis is often challenging and the most widely used diagnostic classifications are the HLH-2004 criteria [8] and the HScore [9], even if significant overlap occurs with common ICU entities such as sepsis [10]. Importantly, early recognition and trigger identification [11] are crucial in improving patient survival. Apart from cause-specific treatment and supportive care, etoposide, a type 2 topoisomerase inhibitor, is the frontline therapy for managing the sickest patients. Etoposide selectively depletes hyperactivated T-CD8 lymphocytes and quickly reverses the cytokine storm [12]. Its efficacy is notorious in pediatric familial HLH [13] and EBV-related HLH [14]. In adult patients, use of first-line etoposide therapy was associated with increased survival in a retrospective study [12].

Healthcare-associated infections (HAIs) are common in the ICU. According to the European Center of Disease Control (ECDC) [15], critically ill patients present with up to 8.3% of at least one HAI. Healthcare-associated pneumonia (HAP) is the most frequently reported (6.3%), followed by bloodstream infections (BSI, 3.7%) and then urinary tract infections (UTIs, 2%). HAIs lead to impaired clinical course with higher mortality rates, prolonged hospital stay [16], as well as increased antimicrobial use, and antimicrobial resistance [15].

To date, data on the rate of HAIs in ICU patients with sHS are scarce. However, sHS patients present a high risk of developing such infections, due to both intrinsic (immunosuppression with specific or drug-induced protracted neutropenia) and extrinsic factors [devices such as endotracheal tube or central venous catheters (CVC)]. In a retrospective study on secondary HS, HAIs were independently associated to worse prognosis [17]. Additionally, whether the administration of early immunosuppressive drugs—namely etoposide—to control macrophage activation increases the risk of HAI in ICU patients is unclear although clinically relevant. The main objective of this study was to assess the influence of etoposide administration on the rate of HAIs in critically ill patients with sHS, and to evaluate whether etoposide is associated with mortality.

## Materials and methods

### Patients, data collection and definitions

We retrospectively included all consecutive adult patients with sHS admitted to the intensive care unit of 1 university hospital from January 2007 to March 2020. There were no exclusion criteria (flowchart, Additional file 1: Figure S1). This study has been approved by the Ethics Committee of the French Intensive Care Society (FICS, no. CE-21-20). In agreement with the French legislation, the database was declared to the CNIL (“Commission Nationale de l’Informatique et des Libertés”) (no. 2221391). We collected epidemiologic, demographic, and medical data, as well as routine blood examinations at the time of ICU admission, including blood count, and serum biochemical tests. HLH diagnosis was established according to the classification developed by the Histiocyte Society in 2004 [8] (Additional file 1: Table S1) and was confirmed jointly by attending hematologists and intensivists. The HScore [9] was calculated from clinical and biological data on admission (Additional file 1: Table S2). Etiological diagnoses were consensually established, according to the results of diagnostic investigations. Since sHS patients may present with more than 1 trigger, final diagnosis was attributed to the cause for which the most clinical evidence existed (Additional file 1: Table S3). Organ failures were defined according to the Sepsis-Related Organ Failure Assessment (SOFA) score which was measured at admission [18]. Prior immunosuppression status was given to any patient with one of the following criteria: HIV infection, cancer or hematologic malignancy ongoing or < 5 years remission, immunosuppressive treatment, solid organ transplant, Castleman’s disease.

Bacterial and fungal infections during ICU stay were recorded and classified as being either: imported from wards HAI (> 48 h after hospital admission but ≤ 48 h after ICU admission) and ICU-acquired HAI (> 48 h after ICU admission). In this study, we focused on patients with any microbiologically documented infection diagnosed after ICU admission, being either imported or acquired in the ICU. Only the first infection was considered for each patient. Following the latest definitions published by the European Center for Disease Prevention and Control (ECDC) [15], HAP was defined in accordance with the PN1 definition with clinical criteria: X-ray imaging, fever > 38 °C or elevated white blood cell count (WBC) > 12000/mm^3^, with new onset of purulent sputum and microbiological confirmation with positive quantitative culture from minimally contaminated lower respiratory tract specimen (≥ 10^4^ colony forming units (CFU)/ml in bronchoalveolar lavage and ≥ 10^3^ CFU/mL in distal protected aspirate, or distal protected brush samples). Bloodstream infections (BSI) were defined as any positive blood culture for a recognized pathogen (and at least 2 blood cultures for skin contaminant pathogens). Urinary tract infections (UTI) were defined as any positive urine culture (≥ 10^5^ microorganisms per ml of urine) associated with fever or UTI symptoms. Central line-associated blood stream infections (CLABSI) were defined as quantitative CVC culture ≥ 10^3^ CFU/mL and pus/inflammation at the insertion site or tunnel or clinical signs improving within 48 h after catheter removal.

### Statistical analysis

Continuous variables are described as median and interquartile range (IQR) and compared using the Kruskal–Wallis test; categorical variables are summarized by counts and frequencies and compared using Fisher’s exact test. Primary endpoint was influence of etoposide on risk of HAI and mortality after double adjustment, more precisely after adjustment for risk factors of the event of interest and propensity score weighting for risk of receiving etoposide. Additional analysis aimed either to be descriptive or to assess robustness of our findings.

Survival analysis was assessed using Kaplan–Meier’s estimates and non-parametric log-rank’s test (Additional file 1: Figure S2). For cumulative incidence of HAIs during ICU stay, we conducted a competing risk survival analysis with the occurrence of HAIs as the primary event of interest and ICU discharge or ICU mortality as time-dependent competing events and comparison performed according to non-parametric Gray’s test [19, 20].

Factors associated with a change in incidence rate of HAIs were assessed using a Fine and Gray subdistribution hazard model [21] and factors associated with mortality were assessed using a time-dependent Cox model. Variable selection was conditional based upon *P* value (entry value *p* < 0.2, exit value *p* > 0.1). Clinically relevant variables not selected in the final model could be forced in as sensitivity analysis. It was planned to force etoposide in the final model should this latter not be selected. Final model was checked for quality, interaction, and adherence to proportional hazard assumption (Additional file 1: Figure S3). Adjustment for variables associated with etoposide use was performed using propensity score-based overlap weighting [22, 23] (Additional file 1: Figure S4). Briefly, overlap weighting was performed. This strategy allows weighting patients from each treatment group with the probability to be assigned the other treatment group. This allows assigning higher weight to patients with intermediate risk and lower weight to outliers in both treatment groups. This analysis emphasizes the part of the population where the most treatment equipoise exists in clinical practice. Propensity score was built using logistic regression according to variables associated with etoposide use and likely to have participated to use of this treatment. Covariates included in the logistic regression model were sHS etiology, HIV infection, severity of the sHS and organ dysfunction at ICU admission. Quality of weighting was assessed using propensity score distribution before and after weighting and variables distribution after weighting. Covariate balance was assessed on the weighted sample using standardized differences (Additional file 1: Figure S4).

Due to limited sample size and to provide accurate estimates of hazard ratios (HR) and their corresponding confidence intervals (95% CI), every one of the provided results reports sHR value and their 95% CI after complex bootstrapping. Briefly, we used a bootstrapping technique, resampling the original set 1000 times with replacement. Then, in each of the sets, we assessed the unadjusted risk, the adjusted risk and last the doubly adjusted weighted risk, for both mortality and cumulative rate of infection. All tests were 2-sided and *p*-values lower than 5% were considered to indicate significant associations. Analyses were performed using R statistical platform, version 3.0.2 and packages *tableone, cmprsk*, *WeightIt, survey,* and *survival.*

## Results

### Demographics and characteristics

During the study period, 168 consecutive patients presenting with secondary hemophagocytic syndrome (sHS) were included. Patients’ characteristics at baseline are summarized in Table 1. Median age was 49 years [38, 59], and most patients were male (*n* = 111, 66.1%). Underlying sHS trigger (Additional file 1: Table S3) was chiefly related to malignancy (*n* = 123, 73.2%), namely B-cell (*n* = 44, 35.8% of malignancies) or T-cell lymphoma (*n* = 38, 30.9%). HS etiology was related to an infection in 25 patients (14.9%), autoimmune disease in 6 patients (3.6%), with only few patients (*n* = 11, 6.5%) having sHS of unknown origin or other causes (*n* = 3, 1.8%). Main comorbidities included HIV infection (*n* = 56, 33.3%), hypertension (*n* = 28, 16.7%), diabetes mellitus (*n* = 14, 8.3%), and chronic kidney disease (*n* = 9, 5.4%). Importantly, most patients had primary or acquired immunosuppression (*n* = 100, 59.5%) prior to developing sHS. Median number of HLH-2004 criteria was 5 [4, 6], with a HScore of 247 [217, 273] and a SOFA score at admission of 8 [6, 12]. Most common HS clinical and biological features included hepatomegaly (*n* = 120, 71.4%), splenomegaly (*n* = 103, 62.4%), bicytopenia (*n* = 132, 78.6%), hyperferritinemia (median level at 10015 µg/L [4980, 32471]), hypertriglyceridemia (3.2 [2.3, 4.3]), histological or cytological features of hemophagocytosis (*n* = 124, 73.8%).

**Table 1:** Overall population characteristics stratified according to etoposide status

	Overall (*n* = 168)	No etoposide (*n* = 33)	Etoposide (*n* = 135)	*p*
Demographics				
Age	49 [38, 59]	49 [36, 59]	49 [38, 60]	0.811
Female gender	57 (34)	14 (42)	43(32)	0.345
Etiology of HS				< 0.001
Malignancy	123 (73.2)	15 (45.5)	108 (80.0)	
Infection	25 (14.9)	13 (39.4)	12 (8.9)	
Auto-immune	6 (3.6)	1 (3.0)	5 (3.7)	
Other	3 (1.8)	0 (0)	3 (2.2)	
Unknown	11 (6.5)	4 (12.1)	7 (5.2)	
Comorbidities				
HIV	56 (33.3)	6 (18.2)	50 (37.0)	0.064
Hypertension	28 (16.7)	11 (33.3)	17 (12.6)	0.009
Diabetes	14 (8.3)	2 (6.1)	12 (8.9)	0.861
Chronic kidney disease	9 (5.4)	4 (12.1)	5 (3.7)	0.135
Immunocompromised	100 (59.5)	17 (51.5)	83 (61.5)	0.397
Features of HLH				
HLH-2004 criteria	5 [4, 6]	4 [4, 5]	5 [5, 6]	0.016
HScore	247 [217, 273]	225 [195, 269]	249 [225, 278]	0.029
Hepatomegaly	120 (71.4)	18 (54.5)	102 (75.6)	0.029
Splenomegaly	103 (62.4)	14 (43.8)	89 (66.9)	0.026
Bicytopenia	132 (78.6)	21 (63.6)	111 (82.2)	0.036
Ferritin (µg/L)	10015 [4982, 32471]	6880 [3705, 18316]	10644 [5297, 40449]	0.025
Fibrinogen (g/L)	3.1 [1.6, 4.9]	3.4 [2.3, 5.1]	2.7 [1.5, 4.8]	0.199
Triglycerides (mmol/L)	3.2 [2.3, 4.3]	3.3 [2.3, 4.3]	3.1 [2.4, 4.3]	0.6
SOFA score	8 [6, 12]	6 [4, 9]	8 [6, 13]	0.008
ICU management				
Mechanical ventilation	91 (54.2)	12 (36.4)	79 (58.5)	0.036
RRT	59 (35.1)	9 (27.3)	50 (37.0)	0.395
Steroids	118 (70.2)	17 (51.5)	101 (74.8)	0.016
Time to etoposide (days)	0 [0, 1]	–	–	–
Outcomes				
HAI	43 (25.6)	2 (6.1)	41 (30.4)	
In-hospital mortality	78 (46.4)	15 (45.5)	63 (46.7)	
Day 90 mortality	83 (49.4)	15 (45.5)	68 (50.4)	0.755

Values are given in N (%) for categorical variables or median [IQR] for continuous variables. Fisher’s exact test for categorical variables, and Kruskal–Wallis test for quantitative variables

*SOFA* Sequential Organ Failure Assessment, *RRT* Renal Replacement Therapy, *ICU* intensive care unit, *HLH* hemophagocytic lymphohistiocytosis, *HIV* Human Immunodeficiency Virus, *HAI* healthcare acquired infection

### ICU management

Overall, 135 patients (80.4%) received etoposide and 118 (70.2%) received steroids in the ICU. Etoposide was mostly administered in the first 24 h after ICU admission (median delay from ICU admission to etoposide administration was 0 days [0, 1]) (Table 1). Additionally, half of patients underwent mechanical ventilation (*n* = 91, 54%), while 81 patients required vasopressors (48%) and 59 (35%) required renal replacement therapy. Forty-eight patients (28.5%) died in the ICU and 78 died in hospital (46.4%) (Additional file 1: Table S4).

In patients who received etoposide, sHS was mostly related to hematological malignancies (Table 1), (*n* = 108, 80% vs. *n* = 15, 45.5%, *p* < 0.001), whereas fewer patients had infection-related HS (*n* = 12, 8.9% vs. *n* = 13, 39.4%, *p* < 0.001). Patients treated with etoposide also presented with increased HScore (median 249, IQR [225, 278] vs. 225 [195, 269], *p* = 0.029), increased number of HLH-2004 criteria (5 [5, 6] vs. 4 [4, 5], *p* = 0.016), higher SOFA score (8 [6, 13] vs. 6 [4, 9], *p* = 0.008), and increased frequency of steroid use (*n* = 101, 74.8% vs. *n* = 17, 51.5%; *p* = 0.016).

### Characteristics of healthcare-associated infections (HAIs)

Forty-three patients (26%) had microbiologically documented HAIs in the ICU, of which 15 (34.9%) were acquired in the wards and 28 (65.1%) in the ICU (Table 2). Overall, infections were mostly of bacterial origin (*n* = 37, 86%), with only 5 fungal infections (11.6%). Infections were more frequently pneumonia (*n* = 14, 32.6%) and primitive bacteremia (BSI) (*n* = 14, 32.6%), followed by urinary tract infection (UTI, *n* = 9, 20.9%), digestive infections (*n* = 4, 9.3%) and CLABSI (*n* = 2, 4.7%). Encountered pathogens were *Pseudomonas aeruginosa* (*n* = 11, 25.6%), *Enterococcus faecium* (*n* = 7, 16.3%), followed by Gram-negative enterobacteria (*Escherichia coli: n* = 4, 9.3%*, Enterobacter cloacae; n* = 3, 7.0*%*), and *Staphylococcus aureus* (*n* = 3, 7%). Time from ICU admission to the first HAI was 4 days [1, 8], while time from etoposide initiation to the first nosocomial event was 5 days [0, 9].

**Table 2:** Characteristics of HAIs occurring after ICU admission

			*n* = 43
Type of infection		Imported nosocomial	15 (34.9)
		In-ICU acquired nosocomial	28 (65.1)
Infection type		Bacterial	37 (86.0)
		Fungal	5 (11.6)
		Both	1 (2.3)
Site of origin		Primary BSI	14 (32.6)
		Pulmonary	14 (32.6)
		UTI	9 (20.9)
		Digestive	4 (9.3)
		CLABSI	2 (4.7)
Positive blood samples			27 (62.8)
Organism cultured	*Gram negative*	*Pseudomonas aeruginosa*	11 (25.6)
		*Escherichia coli*	4 (9.3)
		*Enterobacter cloacae*	3 (7.0)
		*Klebsiella pneumoniae*	2 (4.7)
		*Enterobacter aerogenes*	1 (2.3)
		*Stenotrophomonas maltophilia*	1 (2.3)
		*Comamonas testosteroni*	1 (2.3)
		*Mycoplasma pneumoniae*	1 (2.3)
	*Gram positive*	*Enterococcus faecium*	7 (16.3)
		*Staphylococcus aureus*	3 (7.0)
		*Enterococcus faecalis*	2 (4.7)
		*Streptococcus spp.*	1 (2.3)
	*Fungi*	*Aspergillus spp.*	3 (7.0)
		*Candida glabrata*	2 (4.7)
		*Candida albicans*	1 (2.3)
Time from ICU admission to infection (days)			4 [1, 8]
Time from etoposide to infection (days)			5 [0, 9]

Values are given in N (%) or median [IQR]

*BSI* bloodstream infection, *CLABSI* central line-associated blood stream infection, *UTI* urinary tract infection

### Raw and adjusted influence of etoposide and other factors on risk of HAIs and mortality

**Fig. 1:**
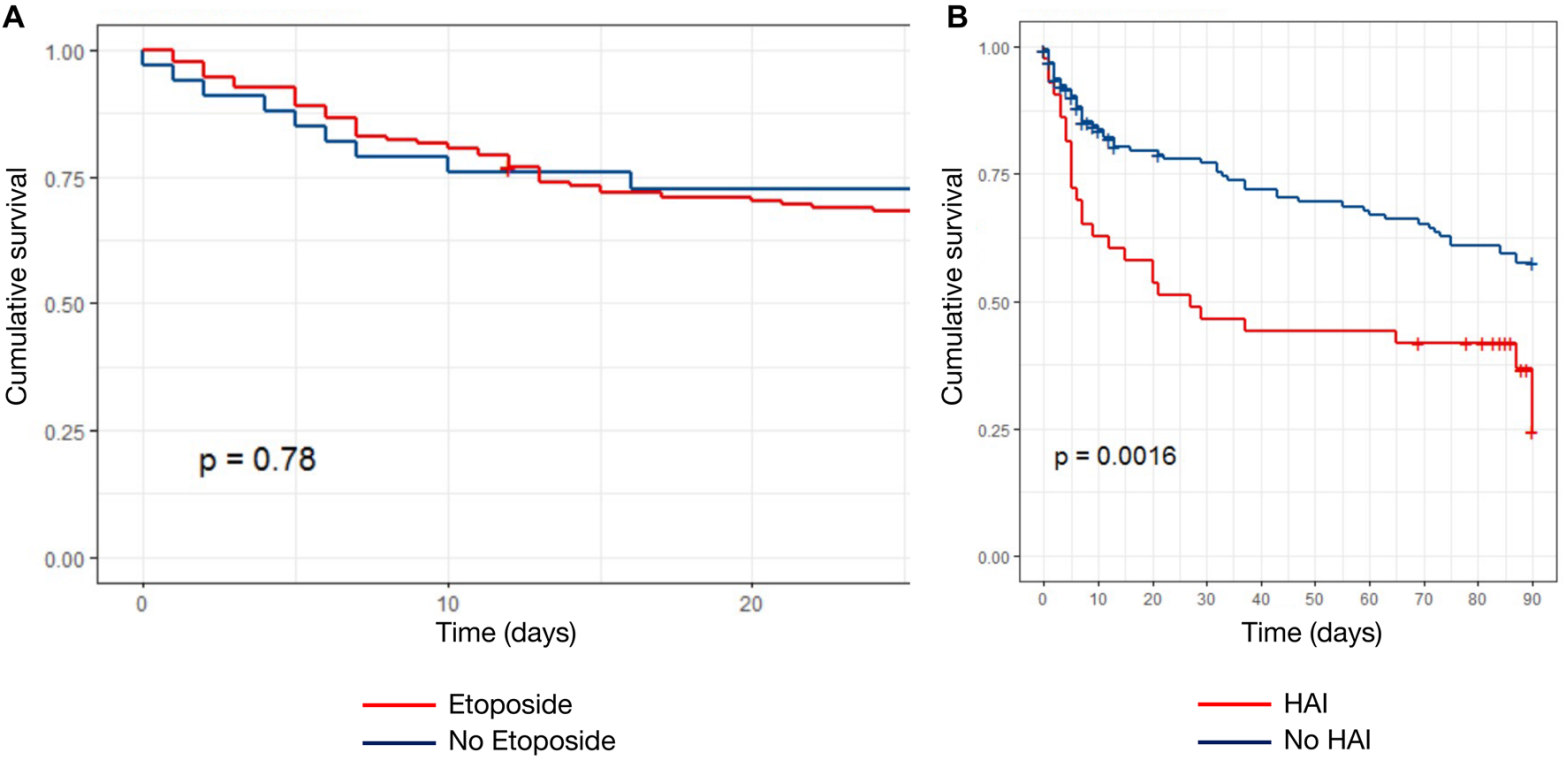
Cumulative survival according to **A** etoposide status and **B** HAIs status (Kaplan–Meier’s estimates, log-rank’s test)

**Fig. 2:**
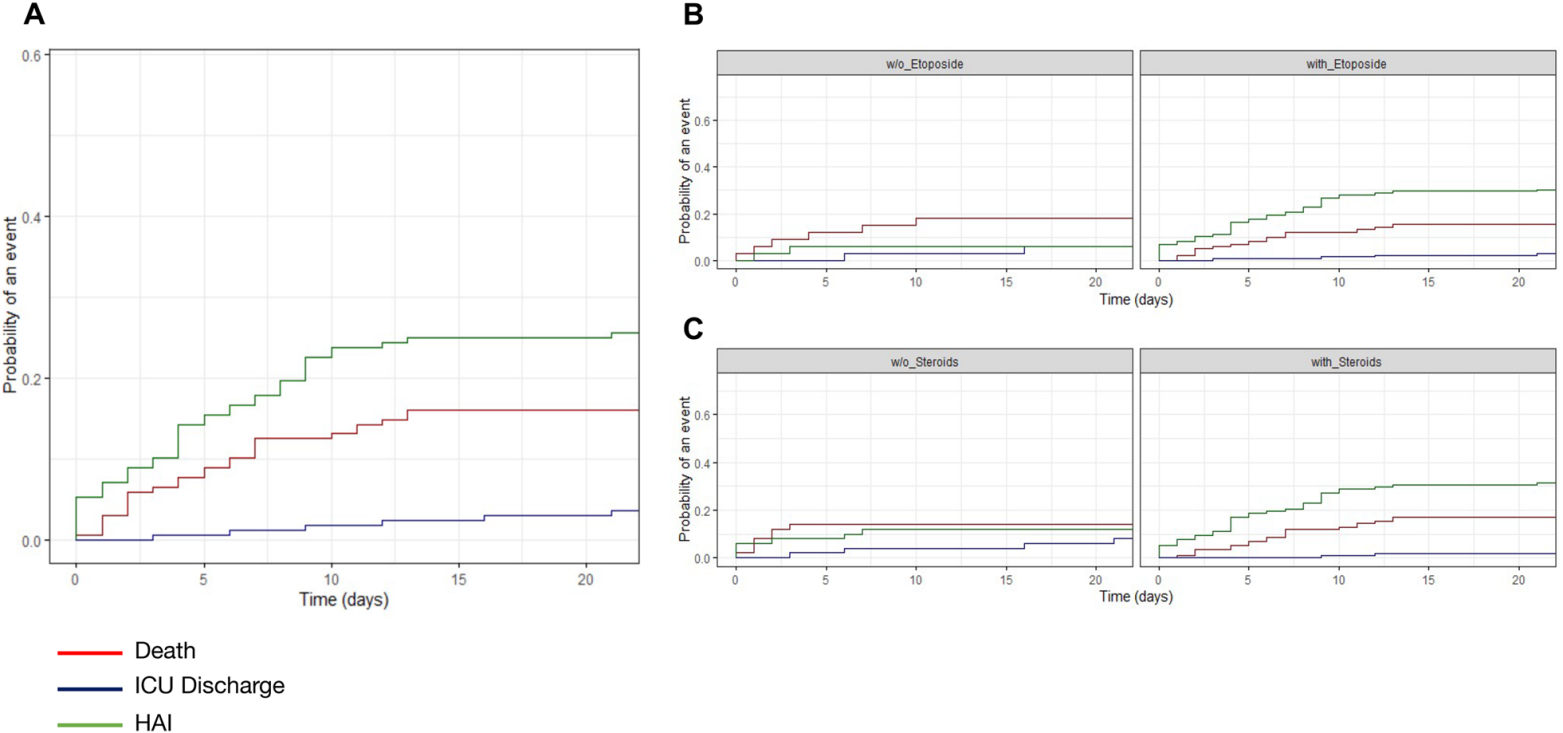
Cumulative incidence functions (CIF) of competing events: HAIs in the ICU, ICU death and ICU discharge: **A** unstratified and stratified according to etoposide (**B)** and steroids (**C**) treatment status

**Fig. 3:**
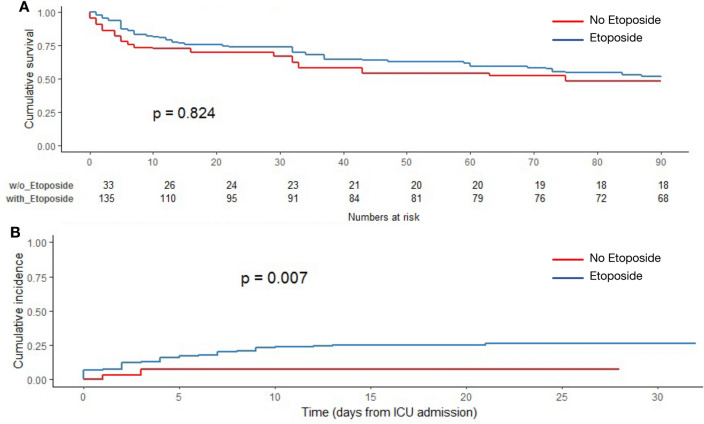
Analysis after propensity score-based overlap weighting **A** Cumulative survival according to etoposide status (Kaplan–Meier estimates, log-rank’s test). **B** Cumulative incidence function (CIF) of HAIs according to etoposide status (Gray’s test)

Etoposide treatment did not affect survival (*p* = 0.78, log-rank) (Fig. 1A), whereas HAIs were associated to a decreased survival rate (*p* = 0.0016, log-rank) (Fig. 1B). In an unadjusted model, etoposide treatment and steroids were both associated with a higher cumulative incidence of HAIs (*p* = 0.005 and *p* = 0.012, Gray’s test) (Fig. 2B, C). Etoposide was associated with risk of HAI before adjustment (sHR 5.95; 95% CI 1.91, 280.4), after adjustment for risk factors of HAI (sHR 5.13; 95%CI 1.6, 151) and after adjustment and weighting (sHR 3.75; 95% CI 1.05–6.67) (Table 3). Elevated SOFA score > 12 (sHR = 2.48 [1.01, 6.12], *p* = 0.048) were associated to an increased rate of HAIs, whereas immune defect was protective (sHR 0.52 [0.28, 0.95], *p* = 0.035) (Table 3). Similarly, cumulative incidence of HAI was higher in patients treated with etoposide (Fig. 3A), while there was no survival benefit of etoposide (Fig. 3B), after weighting. Etoposide was not associated with mortality before adjustment (sHR 0.85; 95%CI [0.36, 2.74]), after adjustment for risk factors of HAI (sHR 0.50; [0.13, 1.92]) and after adjustment and weighting (sHR 0.53; [0.20, 1.98]). Other factors associated with mortality included prior immunosuppression (2.35 [1.35, 4.07], *p* = 0.0028), age (1.02 [1.01, 1.04], *p* = 0.021), SOFA > 13 (4.17 [2.20, 7.91], *p* < 0.0001), HAI (1.94 [1.18, 3.2], *p* = 0.009) (Table 4).

**Table 3:** Factors associated with the incidence of HAI (Fine and Gray proportional hazards model for competing events)

Covariate	sHazard ratio (sHR)—[IC95%]	*p*
Immunodepression	0.52 [0.28, 0.95]	0.035
HScore	1.00 [0.99, 1.01]	0.104
Etoposide treatment	3.75 [1.05, 6.67]	0.032
SOFA score [0–5]	Reference	–
SOFA score [6–8]	3.14 [1.24, 7.9]	0.0157
SOFA score [8–12]	1.71 [0.61, 4.74]	0.31
SOFA score [12–21]	2.48 [1.01, 6.12]	0.048

**Table 4:** Mortality associated risk factors after propensity score-based weighting and multivariate analysis with a Cox proportional hazards model for time-dependent variable after weighting

Covariate	sHazard ratio (sHR)—[IC95%]	*p*
Age	1.02 [1.01, 1.04]	0.021
Male	1.97 [1.12, 3.46]	0.018
Immunodepression	2.35 [1.35, 4.07]	0.0028
SOFA score [0–5]	Reference	–
SOFA score [6–8]	1.28 [0.67, 2.44]	0.448
SOFA score [8–13]	1.78 [0.97, 3.25]	0.062
SOFA score [13–21]	4.17 [2.20, 7.91]	< 0.0001
Hospital acquired infection	1.94 [1.18, 3.2]	0.009

## Discussion

In this cohort of critically ill patients with sHS, using a competing risk survival model, the occurrence of HAIs was associated with decreased cumulative survival. Interestingly, etoposide administration was associated with an increased rate of microbiologically documented HAIs in the ICU, without influencing survival outcomes.

To date, few studies have focused on HAIs in critically ill immunocompromised patients. In a retrospective study, Velasco et al. demonstrated a high rate of HAIs in immunocompromised ICU patients, with an overall rate of 91.7 per 1000 patient-days, with pneumonia, urinary tract infections and bloodstream infections being the most frequent [24]. More recently, in a large cohort of cancer patients, Stoclin et al. reported a cumulative incidence of ventilator-associated pneumonia (VAP) and secondary BSI of 58.8% and 15.1%, respectively, during the first 25 days of exposure [25] and HAIs were not associated with higher ICU mortality. In our cohort, we report a cumulative incidence of HAIs of 25% at day 15 after ICU admission, in the setting of sHS patients. HAIs were associated with a decreased survival rate. Most common causes of in-hospital death included multi-system organ failure (MOF), progressive underlying onco-hematological disease, and acute respiratory distress syndrome (ARDS) (Additional file 1: Table S5). Even though our study found an association between HAIs and increased mortality, final causality of death is hardly solely attributable to HAIs in these patients. In a pooled analysis of 661 ICU patients with sHS, overall mortality was 57.8%, with a higher mortality rate of 63.2% in patients with a malignant trigger [1, 26, 27]. In our study, we report a lower ICU mortality rate of 28.5%. This difference can be explained, in part, by a center effect, since our center is specialized in managing oncohematology patients, with shortened diagnosis and treatment delays which are correlated to better prognosis [11]. We may also hypothesize that sHS patients represent a particular group of patients within the immunocompromised ICU population, presenting with severe organ failures at ICU admission (median SOFA score at 8 [6–12]), explaining why the onset of HAIs is associated with a poorer outcome. Further studies with larger cohorts are needed to confirm our findings.

Also, underlying HIV infection facilitates trigger infections or malignancies thereby causing sHS. In our study, we report a high rate of immunocompromised patients with chronic HIV infection (*n* = 56, 33.3%). This can be explained by a center effect, since our center is specialized in the management of immunocompromised patients, some with acute and chronic HIV infection. There was no sHS triggered by acute HIV infection.

Etoposide constitutes a frontline therapeutic in the management of sHS in the most severe patients, to promptly limit organ failure, as it effectively reverses the cytokine storm by significantly reducing T lymphocyte activation. Early administration of etoposide has shown efficacy in pediatric patients [13, 14]. In adult patients, no randomized study has been conducted. Nevertheless, etoposide was associated with increased survival in a retrospective study [12]. Etoposide may promote the occurrence of HAIs, but data regarding the impact of etoposide on the incidence of HAIs in sHS patients are scarce. In one retrospective study, Apodaca et al. found that HAIs were independently associated with mortality. However, this cohort included a small number of patients and the rate of HAIs was very high (42%) [17]. To our knowledge, this is the first study assessing that the incidence of HAIs is higher in critically ill patients with sHS receiving etoposide. Nevertheless, while HAIs are associated with a poor outcome in the whole population of the study, survival in patients who received etoposide is not impacted. These results may either reflect increased risk of HAI with balanced risk benefit ratio in terms of mortality or a limited statistical power precluding the identification of an association between etoposide and mortality. Such infectious risk should be known to promptly detect and treat infections in these patients with frequent underlying immunosuppression. Current guidelines suggest antiviral, antifungal and pneumocystis prophylaxis for sHS patients needing etoposide administration [28]. Further studies to evaluate the benefit of broader prophylaxis with antibiotics are warranted for these patients.

This study suffers, however, from several limitations. First, this was a single center and retrospective study. Second, the primary event of interest was the first microbiologically documented HAI, which may have led to an underestimation of nosocomial events. Then, even if we used propensity score-based weighting of our population for etoposide exposure, and although groups seemed to be balanced on measured covariates after weighting, we cannot rule out that unmeasured parameters would differ between groups. Among these, EBV replication status, which mostly reflects the depth of immunosuppression, can also influence etoposide response. However, in our cohort, we only reported 4 (2.4%) EBV-associated lymphoproliferations with specific treatment requirements and follow-up. Third, corticosteroids represent a well-known risk factor associated with the occurrence of HAIs. However, we could not analyze this variable in the model, as an interaction exists between steroids and etoposide administration in a sensitivity analysis. Additionally, 25% of HAIs occurred before or on the day of etoposide administration. Since etoposide was administered at ICU admission in most patients, we do not believe that this could affect our results. Finally, as most patients received etoposide in our study, the small sample size of patients who did not received etoposide led to limited statistical power. The lack of benefit of etoposide on survival needs to be interpreted cautiously.

In summary, our study shows that etoposide treatment is associated with a higher incidence of HAIs in the ICU in severe patients with sHS. While HAIs were independently associated with a poor outcome, etoposide administration was not associated with a decreased survival. These results confirm that the use of etoposide to reverse organ failures is feasible assuming an increased risk of nosocomial infection that should be considered and monitored.

## Supplementary Information

**Additional file 1: Figure S1. **Flowchart of the study. **Figure S2** Cumulative survival of all 168 patients (censored at day 90). **Figure S3 **Proportional hazards assumption for Fine and Gray model. **Figure S4 **Covariate balance assessment after inverse probability of treatment weighting. **Table S1 **Revised Diagnostic Guidelines for HLH^1^. **Table S2 **HScore. **Table S3** Detailed causes of hemophagocytic syndrome. **Table S4 **Population characteristics stratified according to in-hospital mortality status (univariate analysis). **Table S5 **Causes of death in patients who died in hospital.

## Data Availability

The datasets used and/or analyzed during the current study are available from the corresponding author upon reasonable request.

## Abbreviations

BSI: Bloodstream infection
CFU: Colony-forming units
CVC: Central venous catheter
CLABSI: Central line-associated blood stream infection
ICU: Intensive care unit
IQR: Interquartile range
HAIs: Healthcare-associated infections
HAP: Healthcare-associated pneumonia
HLH: Hemophagocytic lymphohistiocytosis
HS: Hemophagocytic syndrome
fHS: Familial hemophagocytic syndrome
sHS: Secondary hemophagocytic syndrome
PS: Propensity score
RRT: Renal replacement therapy
SOFA: Sequential Organ Failure Assessment
UTI: Urinary tract infection
