# Supplementary material for: Etoposide treatment in secondary hemophagocytic syndrome: impact on healthcare-associated infections and survival
Source: Ann Intensive Care. 2022 Oct 28;12:101. doi: 10.1186/s13613-022-01075-9 (PMC9616615; doi:10.1186/s13613-022-01075-9)
Supplement: Supplementary file 1 — Additional file 1: Figure S1. Flowchart of the study. Figure S2 Cumulative survival of all 168 patients (censored at day 90). Figure S3 Proportional hazards assumption for Fine and Gray model. Figure S4 Covariate balance assessment after inverse probability of treatment weighting. Table S1 Revised Diagnostic Guidelines for HLH1. Table S2 HScore. Table S3 Detailed causes of hemophagocytic syndrome. Table S4 Population characteristics stratified according to in-hospital mortality status (univariate analysis). Table S5 Causes of death in patients who died in hospital. [file 13613_2022_1075_MOESM1_ESM.docx]

**Etoposide treatment in secondary hemophagocytic syndrome: impact on healthcare-associated infections and survival**

Thibault Dupont MD^1,2^, Michael Darmon MD, PhD^1,2,3^, Eric Mariotte MD^1,2^, Virginie Lemiale MD, PhD^1,2^, Jehane Fadlallah MD ^2, 3, 4^, Adrien Mirouse MD^1,2^, Lara Zafrani MD, PhD^1,2^, Elie Azoulay MD^1,2^, PhD, Sandrine Valade MD^1,2^

1. Assistance Publique-Hôpitaux de Paris (APHP), Medical Intensive Care Unit, Saint-Louis University Hospital, Paris, France
2. Université de Paris, Paris, France
3. ECSTRA team, and Clinical Epidemiology, UMR 1153, Center of Epidemiology and Biostatistics, Sorbonne Paris Cité, CRESS, INSERM, Paris, France.
4. Assistance Publique-Hôpitaux de Paris (APHP), Immunology Department, Saint-Louis University Hospital, Paris, France

**Supplementary material**

- **Figure S1:** Flow chart of the study
- **Figure S2:** Cumulative survival of all 168 patients (censored at day 90)
- **Figure S3:** Proportional hazards assumption for Fine and Gray model
- **Figure S4:** Covariate balance assessment after inverse probability of treatment weighting
- **Table S1:** Revised Diagnostic Guidelines for HLH^1^
- **Table S2:** HScore
- **Table S3:** Detailed causes of hemophagocytic syndrome
- **Table S4:** Population characteristics stratified according to in-hospital mortality status (univariate analysis)
- **Table S5:** Causes of death in patients who died in hospital

**
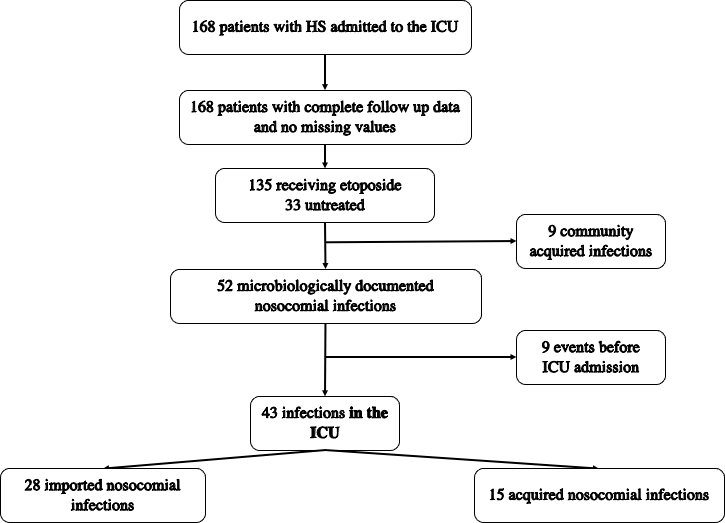
**

**Figure S1:** Flow chart of the study

**
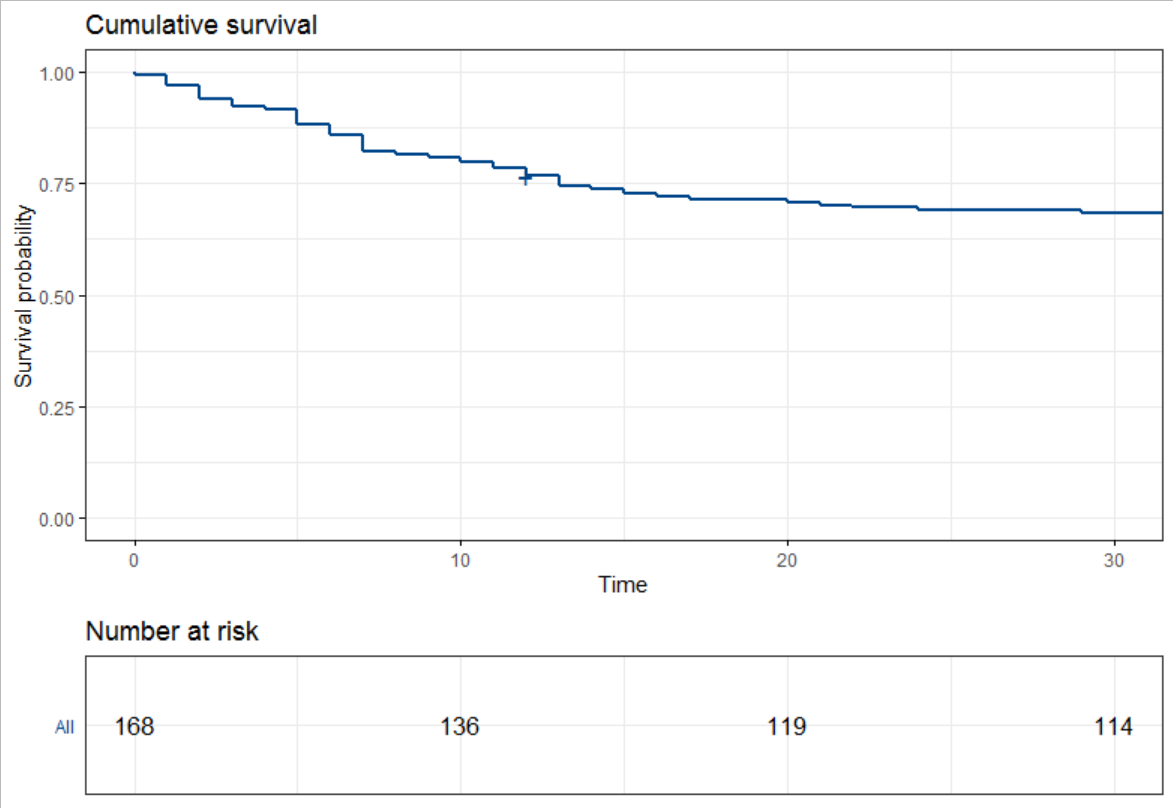
**

**Figure S2:** Cumulative survival of all 168 patients (censored at day 90) using Kaplan-Meier’s estimates.

**
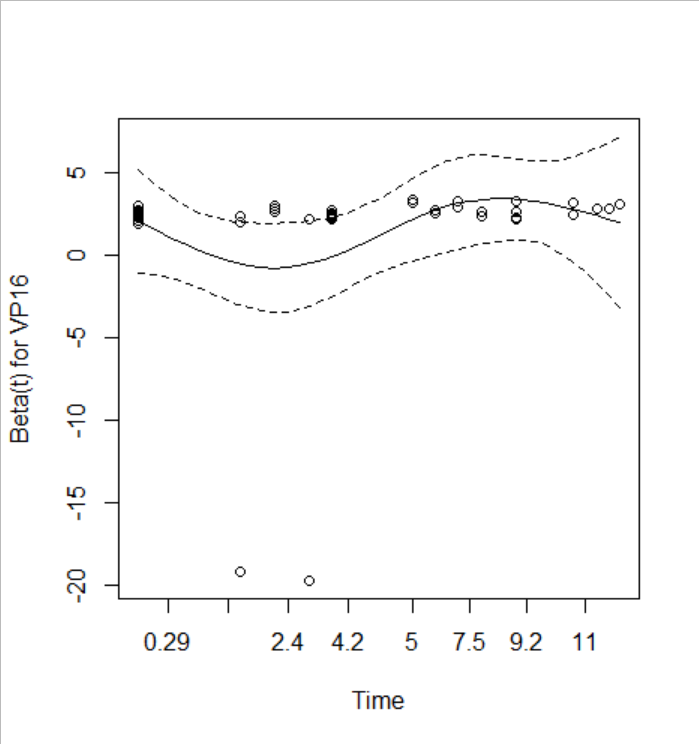
**

**Figure S3:** Proportional hazards assumption for the Fine and Gray model


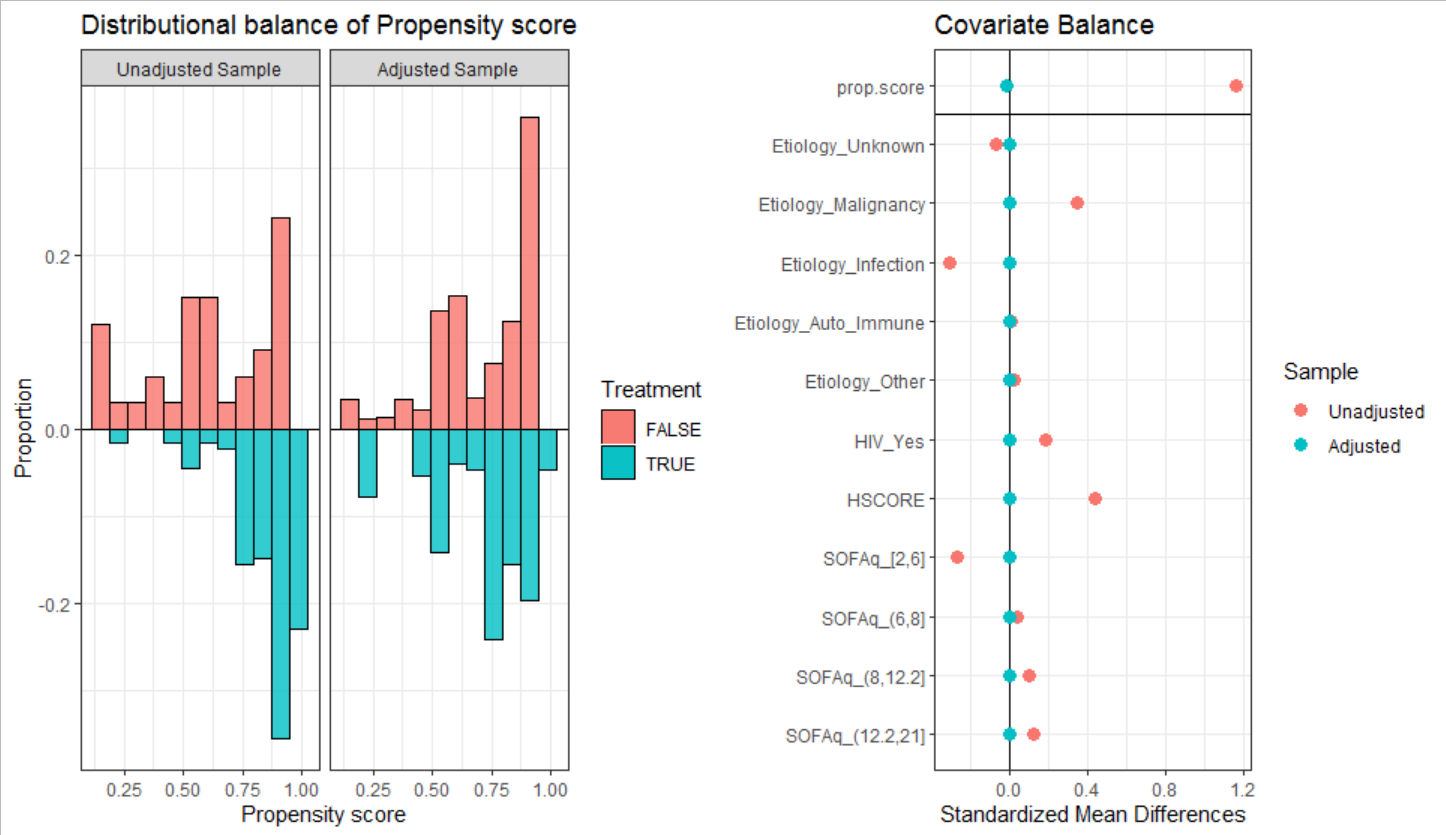


**
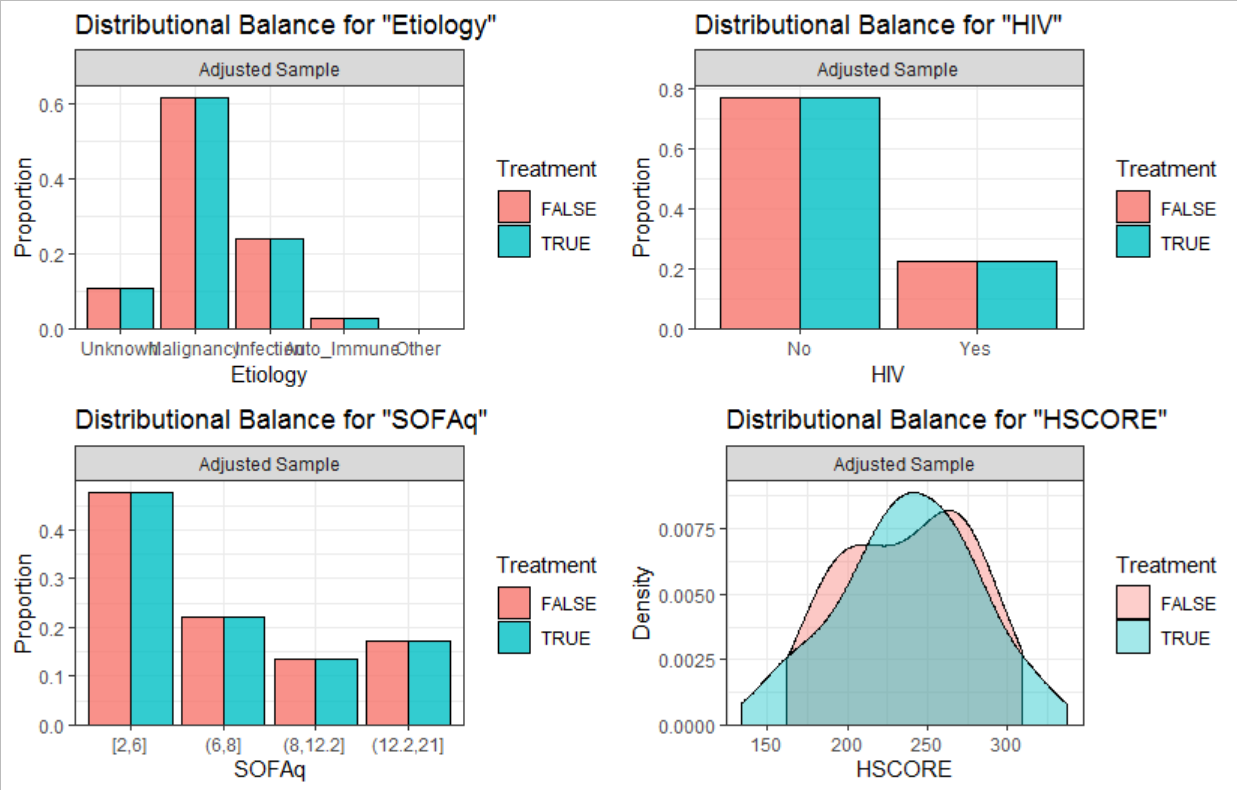
**

**Figure S4**: Covariate balance assessment after propensity score-based overlap weighting **- (A)** Distributional balance of propensity score **(B)** Covariate balance before and after adjustment **(C)** Distributional balance for HS etiology, HIV status, SOFA score, and HScore.

| The diagnosis of HLH can be established if one of either 1 or 2 below is fulfilled  (1) A molecular diagnosis consistent with HLH  (2) Diagnostic criteria for HLH fulfilled (five out of the eight criteria below)  (A) Initial diagnostic criteria (to be evaluated in all patients with HLH)  Fever  Splenomegaly  Cytopenias (affecting ≥ 2 of 3 lineages in the peripheral blood):  Hemoglobin <90 g/L (in infants <4 weeks: hemoglobin <100 g/L) Platelets <100 x 10^9^ /L  Neutrophils <1.0 x 10^9^ /L  Hypertriglyceridemia and/or hypofibrinogenemia: Fasting triglycerides > 3.0 mmol/L (i.e., > 265 mg/dl) Fibrinogen <1.5 g/L  Hemophagocytosis in bone marrow or spleen or lymph nodes  No evidence of malignancy  (B) New diagnostic criteria  Low or absent NK-cell activity (according to local laboratory reference)  Ferritin > 500 mg/L  Soluble CD25 (i.e., soluble IL-2 receptor) > 2400 UI/ml |
| --- |

**Table S1:** Revised Diagnostic Guidelines for HLH^1^. Abbreviations: HLH: Hemophagocytic Lymphohistiocytosis

| **Parameter** | **No. of points (criteria for scoring)** |
| --- | --- |
| Known underlying immunosuppression (Human immunodeficiency virus positive or receiving long‐term immunosuppressive therapy (i.e., glucocorticoids, cyclosporine, azathioprine). | 0 (no) or 18 (yes) |
| Temperature (°C) | 0 (<38.4), 33 (38.4–39.4), or 49 (>39.4) |
| Organomegaly | 0 (no), 23 (hepatomegaly or splenomegaly), or 38 (hepatomegaly and splenomegaly) |
| No. of cytopenias (hemoglobin level of ≤9.2 gm/dl and/or a leukocyte count of ≤5,000/mm3 and/or a platelet count of ≤110,000/mm3) | 0 (1 lineage), 24 (2 lineages), or 34 (3 lineages) |
| Ferritin (ng/mL) | 0 (<2,000), 35 (2,000–6,000), or 50 (>6,000) |
| Triglycerides (mmol/L) | 0 (<1.5), 44 (1.5–4), or 64 (>4) |
| Fibrinogen (g/L) | 0 (>2.5) or 30 (≤2.5) |
| Serum glutamic oxaloacetic transaminase (UI/L) | 0 (<30) or 19 (≥30) |
| Hemophagocytosis features on bone marrow aspirate | 0 (no) or 35 (yes) |

**Table S2A**

| **HScore** | **Probability of hemophagocytic syndrome** |
| --- | --- |
| 90 | 3 |
| 100 | 5 |
| 110 | 9 |
| 120 | 16 |
| 130 | 25 |
| 140 | 40 |
| 150 | 54 |
| 160 | 70 |
| 170 | 80 |
| 180 | 88 |
| 190 | 80 |
| 200 | 88 |
| 210 | 93 |
| 220 | 96 |
| 230 | 98 |
| 240 | 99 |
| 250 | >99 |

**Table S2B**

**Table S2: (A)** Composition of the HScore (**B**) Attached probability of hemophagocytic syndrome. NB: The best cutoff value for HScore was 169, corresponding to a sensitivity of 93%, a specificity of 86%, and accurate classification of 90% of the patients. (adapted from Fardet et al.^2^)

| **Underlying HS trigger** |  | **N (%)** |
| --- | --- | --- |
| **Onco-hematology** |  | **123 (73.2)** |
| T-cell lymphoma |  | 38 (30.6) |
| B-cell lymphoma |  | 44 (35.5) |
| Hodgkin lymphoma |  | 17 (13.7) |
| Multicentric Castleman disease/Kaposi sarcoma |  | 19 (15.4) |
| Other |  | 5 (4.8) |
| **Infectious** |  | **25 (14.9)** |
| Viral |  | 6 (22) |
| Mycobacteria |  | 10 (37) |
| Parasite |  | 5 (19) |
| Fungi |  | 6 (22) |
| **Autoimmune** |  | **6 (3.6)** |
| Lupus |  | 1 (0.6) |
| Still's disease |  | 5 (3.6) |
| **Other** |  | **3 (1.8)** |
| **Unknown origin** |  | **11 (6.5)** |

**Table S3:** Detailed causes of hemophagocytic syndrome

|  | **Overall (n = 168)** | **Hospital discharge (n = 90)** | **In hospital mortality (n = 78)** | **p** |
| --- | --- | --- | --- | --- |
| **Demographics** |  |  |  |  |
| Age | 49 [38, 59] | 44 [35, 54] | 52.00 [41, 64] | 0.005 |
| Female gender | 57 (34) | 54 (60) | 57 (73) | 0.105 |
| **Etiology** |  |  |  | 0.451 |
| Malignancy | 123 (73.2) | 66 (73.3) | 57 (73.1) |  |
| Infection | 25 (14.9) | 13 (14.4) | 12 (15.4) |  |
| Auto-immune | 6 (3.6) | 5 (5.6) | 1 (1.3) |  |
| Other | 3 (1.8) | 2 (2.2) | 1 (1.3) |  |
| Unknown | 11 (6.5) | 4 (4.4) | 7 (9.0) |  |
| **Comorbidities** |  |  |  |  |
| HIV | 56 (33.3) | 32 (35.6) | 24 (30.8) | 0.623 |
| Hypertension | 28 (16.7) | 9 (10.0) | 19 (24.4) | 0.022 |
| Diabetes Mellitus | 14 (8.3) | 6 (6.7) | 8 (10.3) | 0.576 |
| Chronic Kidney Disease | 9 (5.4) | 3 (3.3) | 6 (7.7) | 0.364 |
| Immunocompromised | 100 (59.5) | 49 (54.4) | 51 (65.4) | 0.199 |
| **Features of HLH** |  |  |  |  |
| HLH-2004 criteria | 5 [4, 6] | 5 [4, 5] | 5 [5, 6] | 0.052 |
| HScore | 247 [217, 273] | 242 [217, 268] | 256 [218, 283] | 0.062 |
| Hepatomegaly | 120 (71.4) | 66 (73.3) | 54 ( 69.2) | 0.678 |
| Splenomegaly | 103 (62.4) | 57 (65.5) | 46 ( 59.0) | 0.481 |
| Bicytopenia | 132 (78.6) | 68 (75.6) | 64 ( 82.1) | 0.404 |
| Ferritin (µg/L) | 10015 [4982, 32471] | 8656 [4319, 2271] | 14273 [5848, 39390] | 0.098 |
| Fibrinogen (g/L) | 3.1 [1.6, 4.9] | 3.74 [1.78, 4.92] | 2.46 [1.40, 4.72] | 0.16 |
| Triglycerides (mmol/L) | 3.2 [2.3, 4.3] | 3.1 [2.3, 4.2] | 3.4 [2.4, 4.7] | 0.29 |
| **ICU management** |  |  |  |  |
| SOFA score | 8 [6, 12] | 7 [5, 10] | 10 [7, 15] | <0.001 |
| Mechanical ventilation | 91 (54.2) | 34 (37.8) | 57 ( 73.1) | <0.001 |
| RRT | 59 (35.1) | 17 (18.9) | 42 ( 53.8) | <0.001 |
| Etoposide/VP16 treatment | 135 (80.4) | 72 (80.0) | 63 ( 80.8) | 1 |
| Corticosteroids | 118 (70.2) | 59 (65.6) | 59 ( 75.6) | 0.209 |
| **Outcomes** |  |  |  |  |
| Nosocomial infections | 43 (25.6) | 17 (18.9) | 26 ( 33.3) | <0.001 |
| In hospital death | 78 (46.4) | 0 (0.0) | 78 (100.0) | <0.001 |
| Day 90 mortality | 83 (49.4) | 6 (6.7) | 77 (98.7) | <0.001 |

**Table S4: Overall population characteristics stratified according to in hospital mortality status.** Values are given in N (%) for categorical variables or median [IQR] for continuous variables. Fisher’s exact test for categorical variables, and Kruskal Wallis’ test for quantitative variables. Abbreviations: SOFA = Sequential Organ Failure Assessment; RRT: Renal Replacement Therapy; ICU: intensive care unit; HLH: Hemophagocytic lymphohistiocytosis; HIV: Human Immunodeficiency Virus.

**Table S5**: Cause of death for patients who died in hospital (n = 78)

| **Cause of death (n = 78)** | **Count (%)** |
| --- | --- |
| Multiorgan failure | 45 (57.7) |
| Progression of underlying malignancy | 13 (16.7) |
| Acute respiratory distress syndrome | 5 (6.4) |
| Intracerebral hemorrhage | 4 (5.1) |
| Invasive fungal infection | 4 (5.1) |
| Cerebral death | 2 (2.6) |
| Cardiac arrest | 1 (1.3) |
| Refractory status epilepticus | 1 (1.3) |
| Hemorrhagic shock | 1 (1.3) |
| Not specified (missing) | 2 (2.6) |

**References**

1. Henter J-I, Horne A, Aricó M, et al. HLH-2004: Diagnostic and therapeutic guidelines for hemophagocytic lymphohistiocytosis. *Pediatr Blood Cancer*. 2007;48(2):124-131. doi:10.1002/pbc.21039

2. Fardet L, Galicier L, Lambotte O, et al. Development and Validation of the HScore, a Score for the Diagnosis of Reactive Hemophagocytic Syndrome. A*rthritis & Rheumatology*. Published September 1, 2014. Accessed December 30, 2020. http://onlinelibrary.wiley.com/doi/abs/10.1002/art.38690
